# A cooperative function for multisensory stimuli in the induction of approach behavior of a potential mate

**DOI:** 10.1371/journal.pone.0174339

**Published:** 2017-03-17

**Authors:** Anders Ågmo, Eelke M. S. Snoeren

**Affiliations:** Department of Psychology, UiT the Arctic University of Norway, Tromsø, Norway; University of Texas at Austin, UNITED STATES

## Abstract

Intrasexual competition is an important element of natural selection in which the most attractive conspecific has a considerable reproductive advantage over the others. The conspecifics that are approached first often become the preferred mate partners, and could thus from a biological perspective have a reproductive advantage. This underlines the importance of the initial approach and raises the question of what induces this approach, or what makes a conspecific attractive. Identification of the sensory modalities crucial for the activation of approach is necessary for elucidating the central nervous processes involved in the activation of sexual motivation and eventually copulatory behavior. The initial approach to a potential mate depends on distant stimuli in the modalities of audition, olfaction, vision, and other undefined characteristics. This study investigated the role of the different modalities and the combination of these modalities in the sexual incentive value of a female rat. This study provides evidence that the presence of a single-sensory stimulus with one modality (olfaction, vision, or ‘others’, but not audition) is sufficient to attenuate the preference for a social contact with a male rat. However, a multisensory stimulus of multiple modalities is necessary to induce preference for the stimulus over social contact to a level of an intact receptive female. The initial approach behavior, therefore, seems to be induced by the combination of at least two modalities among which olfaction is crucial. This suggests that there is a cooperative function for the different modalities in the induction of approach behavior of a potential mate.

## Introduction

Natural selection is one of the key components of evolution theory. Charles Darwin described it as the differential survival and reproduction of individuals due to differences in phenotype. In order to have reproductive success, individuals must be more attractive within a population or preferring more attractive partners to produce offspring. While intrasexual competition for access to a mate is believed to be common among mammals, competition is unusual in wild rats [1, 2]. Though, even if intrasexual competition were unusual, rats still have to make a choice of partner with whom to initiate copulatory activity whenever there is more than one potential partner available.

In laboratory studies, it was shown that the first approached partner in a multiple partner paradigm was the rat with whom most sexual interactions occurred [3–5]; a result that was found in both males and females. These rats could, from a biological perspective, have a considerable reproductive advantage over others. This underlines the importance of the initial approach and raises the question of what induces this approach, or what makes a conspecific attractive.

Since approach to a potential mate must depend on distant stimuli, the sensory modalities involved in mate choice can be audition (via ultrasonic vocalizations (USVs)), olfaction (the smell of a receptive female), or vision (the sight of a receptive female). It has been shown that visual stimuli are not important, since males approach females even in complete darkness [6, 7]. Olfactory stimuli, on the contrary, are important for the incentive value of females. Intact male rats approach the odor of sexually receptive females more than they approach non-receptive females [8–11], while anosmic males loose their capacity to distinguish between females in estrus or non-estrus [12]. The roles of the auditory stimuli are less clear. However, rats emit 50 kHz ultrasonic vocalizations (USVs) in the presence of a sexual partner and during copulation [13–15], but previous studies have shown that these USV do not have any incentive value for male and female rats [10, 16].

The option that one single-sensory modality does not induce approach behavior by itself does not necessarily mean that this modality does not play a role in the incentive value of the female as a whole. In a natural situation, a female is presented as ‘the combination of several modalities’ at the same time. The investigation of a single modality does, therefore, not represent the conditions in nature. It could, for example, be possible that one modality has no incentive value by itself but might add value to another modality, thereby making the rat more attractive as potential mate. This strengthens the need for research on the potential cooperative function for the different modalities in the induction of approach behavior of a potential mate.

In addition, identification of the sensory modality or combination of modalities crucial for the activation of approach to a sexually relevant stimulus is necessary for elucidating the central nervous processes involved in the activation of sexual motivation and eventually copulatory behavior in the recipient of the stimulus. We already know that gonadal hormone actions in the medial preoptic area and the ventromedial nucleus of the hypothalamus are essential for the activation of sexual approach and copulation in males and females [17–20], respectively. However, we do not know exactly how sexually relevant stimuli alter the neural activity in these areas, partly because the nature of these stimuli remain unclear. Furthermore, it is not possible to understand the bases for interindividual variations in sexual attractivity, which is an important determinant of reproductive success, unless we know which stimulus modality or combination of modalities is involved in the incentive value of a conspecific. In many non-rodent species, ethologists have described these stimuli [21, 22], but in rodents they are far less known. This is unfortunate, because many of neurobiology’s most powerful techniques are easily available for rodents but less so for most other species. It is thus crucial to identify the attractive component(s) of a sexually relevant stimulus in order to elucidate the central nervous processes involved in the approach of a potential mate, possibly leading to reproductive success.

In addition, the suggestion that the modalities involved in approach behavior could only be vision, olfaction or audition (as in USVs) is not completely true. There could be other factors involved that are not defined yet. Characteristics that could be involved and are so far neglected, are for example the sound of a moving or darting female, the smell of a rat being closer or more distant (besides the smell of receptivity), or the feeling of a temperature change or a flow in the air induced by a moving rat, and probably other factors that we cannot immediately think of but could exist. We, therefore, introduce another ‘modality’ in our study that we from now on call ‘others’. Even though the ‘others’ can not be described as a modality or a single-sensory stimulus, we put them in such category. Nevertheless, that category should be considered as the combination of those stimuli that (so far) have not been distinguished.

In this study we investigated the role of the different modalities and the combination of these modalities in the incentive value of a female rat. What single-sensory stimulus or combination of stimuli (multisensory) is required to induce approach behavior in males? We provide evidence that olfaction is necessary, but visual stimuli or ‘other’ need to be added to olfaction in order to induce preference for the stimulus over social contact with another male rat.

## Materials and methods

### Ethics statement

All experimentation was carried out in agreement with the European Union council directive 2010/63/EU. The protocol was approved by the National Animal Research Authority in Norway. Isoflurane anesthesia was used for the rats that underwent ovariectomy or devocalization. The anosmia procedure, on the other hand, was performed under ketamine/xylazine anesthesia (100 mg/kg and 10 mg/kg, respectively). All rats that underwent surgery were treated with buprenorphine (.05 mg/kg subcutaneously) at surgery and again every 12 hours for the following 3 days, and all efforts were made to minimize suffering.

### Subjects

In total, one hundred and sixty-three experimental male rats (weighing 300 g at the start of the experiment), two stimulus male rats (300 g), and eighteen stimulus female rats (250 g) were used in this experiment. All Wistar rats were obtained from Charles River (Sulzfeld, Germany). The rats were housed in same-sex pairs in Macrolon IV® cages on a reversed 12 hours light/dark cycle (lights on 23:00–11:00), in a room with controlled temperature (21±1°C) and relative humidity (55±10%). Standard rodent food and tap water were available ad libitum.

### Reagents

The females were subcutaneously implanted with a 5 mm long Silastic capsule (medical grade Silastic tubing, .0625 in. inner diameter, .125 in.outer diameter, Degania Silicone, Degania Bet, Israel) containing 10% 17β-estradiol in cholesterol (Sigma, St. Louis, MO, USA). The ends of the capsules were sealed with medical grade adhesive silicone (Nusil Silicone Technology, Carpinteria, CA USA). Before every behavioral test, the females were given progesterone (Sigma, St Louis, MO, USA) in a dose of 1 mg/rat approximately 4 hours prior to testing to induce receptivity. The steroid was dissolved in peanut oil (Apoteksproduskjon, Oslo, Norway) and injected subcutaneously in a volume of .2 ml/rat.

Anosmia was induced by bilateral intranasal infusions of 10% Zinc sulfate heptahydrate (ZnSO_4_) in 0.9% saline in a volume of 100 μl (Sigma, St. Louis, MO, USA).

All rats that underwent surgery were treated with buprenorphine (.05 mg/kg subcutaneously) at surgery and again every 12 hours for the following 3 days.

### Surgery procedures

#### Ovariectomy

All female rats were ovariectomized under isoflurane anesthesia at least 2 weeks before use and implanted with the Silastic hormonal capsule. This was done with a 1-cm dorsal incision of the skin and 2 small incisions in the muscles on both sides to reach the ovaries. The ovaries were extirpated and the capsule was placed subcutaneously in the same incision, before the muscles and skin were sutured.

#### Devocalization

Two female rats were devocalized under isoflurane anesthesia at least 2 weeks before use. A 2-cm incision on the ventral surface of the neck was made, followed by the separation of the sternohyoideus muscles to expose the trachea and locate the recurrent laryngeal nerves. The nerve was freed from the surrounding fascia, lifted up and a section of about 3 mm of the nerve was removed bilaterally. The incision was closed with subcutaneous sutures.

At the time of use, the females were tested for the absence of ultrasonic vocalizations with a procedure previously described [16]. This test was conducted in a four-chamber cage, in which the middle cage was attached to 3 round cages (diameter of 50 cm) connected by a wire mesh, above which a high frequency sensible microphone (obtained from Metris, Hoofddorp, The Netherlands) was placed. The microphone was connected to a computer with the Sonotrack® sound analysis system. The (hormonally primed) females were placed in the attached cages, while a male rat was situated in the middle cage, and the vocalizations were recorded for 5 minutes. When females are intact, they produce USVs in this situation. The devocalized females in this experiment, however, did not emit any USVs, which proved that they were successfully devocalized.

#### Anosmia

The anosmia was induced three days before the sexual incentive motivation test. The surgery was performed under ketamine/xylazine anesthesia (100 mg/kg and 10 mg/kg, respectively). The anesthetized males were placed on their back on an inclined surface with their heads facing downwards. A plastic tube was gently placed in each nostril and 100 μl ZnSO_4_ was slowly infused in each nasal cavity over a period of 1 minute. The tubes were left in place for another minute before being removed. During the whole procedure and during recovery, the mouth of the rat was aspirated to remove saliva and excess solution to prevent swallowing that could lead to sickness or death.

A few hours before the sexual incentive motivation test, the anosmic rats were tested for the success of the anosmia induction by a chocolate pellet test. Before the anosmia induction, the rats were already introduced to chocolate pellets. During the chocolate pellet test, the rats were placed in another cage in which 3 chocolate pellets were hidden under the bedding. Intact males find the chocolate pellets within 1 minute. When a ZnSO_4_-treated male did not find a pellet within 5 minutes, the anosmia induction was considered successful. Only males that were anosmic were used in this experiment.

### Apparatus

The incentive value of the multisensory stimuli was tested in a sexual incentive motivation test. This test was performed in a rectangular arena (100 x 50 x 45 cm) with rounded corners. The walls consisted of metal sheet covered with a black plastic surface and the floor was made of dark-gray polyvinyl chloride. At the long sides, 15 cm from opposite corners, there were openings (25 x 25 cm) linked to two incentive stimulus cages connected from the outside of the observation arena. The incentive stimulus was separated from the experimental male by a wire mesh. Outside each incentive stimulus cage, a virtual zone of 30 x 21 cm was defined. The experimental male was considered to be within the zone whenever its point of gravity was inside. Most tests were performed in a room that was illuminated with dim white light, about 5 lx at the bottom of the arena. The few tests in which vision was excluded from the multisensory stimuli were conducted in complete darkness. Two infrared lamps (850 nm; model Sal-60. New Surway Digital Technology (Shenzhen), Guangdong, P.R. China) assured that the high resolution, digital B/W camera (JVJ-331H) produced a clear image of the arena. The video camera located in the ceiling above the observation arena was connected to a computer and a video tracking system (Ethovision XT, Noldus, Wageningen, The Netherlands). The experimental male’s position was determined with a frequency of 5 Hz. The program calculated the time the experimental males spent in each incentive zone, the distance moved during the test, the mean velocity of movement, and the time moving [23, 24].

### Auditory stimuli recording

An USV recording from a previous experiment was used as auditory stimulus, which will be called ‘series of calls’ in this manuscript. The procedure of the recording of the ‘full song I’ can be found under “devocalization”. More details about the calls in the stimulus are available in [10].

### Procedure

Two weeks before the sexual incentive motivation test, the male rats obtained sexual experience in a copulation cage on 3 occasions (separated by 48 hours) in which they were allowed to copulate with a hormonally primed female until the first ejaculatory series was completed. One week prior to the experiment, the males were familiarized to the observation arena during 3 sessions of 10 minutes each, separated by 48 hours. During these sessions, the incentive stimulus cages were empty. The week after, the sexual incentive motivation (SIM) tests were performed.

Before each experimental session the arena was carefully cleaned with a .1% solution of glacial acetic acid in water. At tests, a stimulus male rat was placed in one incentive cage, while the other cage contained one of the different stimuli. At the beginning of the test, the experimental male was introduced into the middle of the arena and the 10 minutes of observation was started immediately. After the test, the experimental male was removed, and the following rat was immediately introduced. The position of the incentive stimuli was semi-randomly changed throughout the experimental sessions.

### Design

In this study, we investigated what stimulus (single-sensory) or what combination of stimuli (multisensory) is required to induce approach behavior in males. Male rats (n = 9–12) were placed in a sexual incentive motivation test for 10 minutes and presented with two incentives: a male rat (as a social ‘control’ situation) and ‘the stimulus’. As stimulus, the four modalities audition, olfaction, vision, and ‘others’ were presented alone (single-sensory stimulus) or in different combination as multisensory stimulus. All possible combinations of stimuli were presented in a semi-randomized order. A between-subject design was used, meaning that one male was only presented with one type of stimulus. This design was chosen because some rats (that were not presented with the modality olfaction) needed to be made anosmic. Therefore, a within-subject design could not be used.

### The different (multisensory) stimuli

Each combination of modalities that was used as stimulus required a different approach. Each (multisensory) stimulus is described in Table 1.

1. *Zero modalities*. As control condition, an empty incentive cage was presented together with an incentive cage containing a (stimulus) male rat. The experimental rat was a male rat. The SIM test was performed under light conditions of 5 lux.
2. *One modality; audition*. When the approach behavior towards only the USVs were investigated, an experimental male was presented with a (stimulus) male in one incentive cage and a loudspeaker from Sonotrack® (50 W high-end ultrasound speaker (from 20 Hz to 150 kHz) obtained from Metris, Hoofddorp, the Netherlands) was placed in the other incentive cage. The loudspeaker played back a series of calls (50 kHz USV) that were recorded previously [10] during the complete 10 minutes test. More details about the type of calls in this series of calls are described in Table 1 (Full song I) of [10]. The SIM test was performed under light conditions of 5 lux.
3. *One modality; olfaction*. Six hours before the start of the SIM test, a hormonally primed female rat was placed in an incentive cage. Immediately before the test, the female was removed from the cage, leaving the olfactory stimulus in the incentive cage (consisting of urine and feces from the female). This incentive cage was used as stimulus in the SIM versus a cage with a (stimulus) male rat. (The successfulness of this olfactory stimulus was previously shown in Snoeren et al. (2013) [10]. A male was used as experimental rat. The SIM test was performed under light conditions of 5 lux.
4. *One modality; vision*. Again, an anosmic male was used as experimental rat. He was presented with an anaesthetized (hormonally primed) female in one incentive cage and a (stimulus) male in the other incentive cage. The SIM test was performed under light conditions of 5 lux. (Anesthetized females do not produce any USVs, which was also confirmed before the experiments.)
5. *One modality; ‘others’*. To remove the modality olfaction from this stimulus, an anosmic male was used as experimental rat. This experimental rat was able to approach an incentive cage with a (stimulus) male on one side and a devocalized (hormonally primed) female on the other side. To remove the modality vision as well, the SIM test was performed in complete darkness.
6. *The two modalities audition and olfaction*. In the SIM test, an experimental male was presented with the odor of a hormonally primed female rat (see under 3) in combination with a loudspeaker playing back the ‘series of calls’ (see under 2) in one incentive cage. A (stimulus) male rat was used in the other incentive cage. The SIM test was performed under light conditions of 5 lux.
7. *The two modalities audition and vision*. Again, an anosmic male was used as experimental rat in the SIM test. He was presented with a (stimulus) male in one incentive cage and an anesthetized (hormonally primed) female in combination with a loudspeaker playing back the ‘series of calls’ in the other incentive cage. The test was performed under light conditions of 5 lux.
8. *The two modalities audition and ‘others’*. When this stimulus was presented, an anosmic male was used as experimental rat. The incentive cages consisted of a (stimulus) male or a hormonally primed female, and the SIM test was performed under complete darkness.
9. *The two modalities olfaction and vision*. A male was used as experimental rat in the SIM test that was performed under light conditions of 5 lux. A (stimulus) male rat was placed in one incentive cage and an anesthetized (hormonally primed) female in the other. This way, the experimental rat can see and smell a female who is not moving or emitting USVs.
10. *The two modalities olfaction and ‘others’*. In the SIM test, an experimental male was presented with the stimulus consisting of a devocalized (hormonally primed) female compared to a (stimulus) male rat. The test was performed under complete darkness to remove the modality vision from the multisensory stimulus.
11. *The two modalities vision and ‘others’*. In the SIM test, an anosmic male is used as experimental rat. He was presented with a devocalized (hormonally primed) female in one incentive cage and a (stimulus) male in the other. The test was performed under light conditions of 5 lux.
12. *The three modalities audition*, *olfaction*, *and vision*. An experimental male was tested with a (stimulus) male in one incentive cage versus an anesthetized (hormonally primed) female in combination with the playback of USV in the other incentive cage. This was done under light conditions of 5 lux.
13. *The three modalities audition*, *olfaction*, *and ‘others’*. An experimental male was presented with a (stimulus) male rat and hormonally primed female rat in each incentive cage. This was done under complete darkness.
14. *The three modalities audition*, *vision*, *and ‘others’*. Since this stimulus did not contain the modality olfaction, an anosmic male was used as experimental rat. He was presented to a (stimulus) male rat in one incentive cage, and a hormonally primed female in the other incentive cage. This was done under light conditions of 5 lux.
15. *The three modalities olfaction*, *vision*, *and ‘others’*. In the SIM test, an experimental male rat was presented with a (stimulus) male in one incentive cage, and a devocalized (hormonally primed) female in the other incentive cage. This was done under light conditions of 5 lux.
16. *The four modalities presented together*. When all modalities were presented as one multisensory stimulus, a male rat was used as experimental rat. In the SIM test, he was presented with a hormonally primed female compared to a (stimulus) male in the incentive cages. This was done under light conditions of 5 lux.

**Table 1:** Experimental design of the study.

Stimulus group	Modality				Experimental male	Incentive cages		Light condition
	Audition	Olfaction	Vision	Others		Stimulus	Control	
1 (n = 11)	-	-	-	-	Male	Empty cage	Male	5 lux
2 (n = 10)	+	-	-	-	Male	Playback of USV	Male	5 lux
3 (n = 11)	-	+	-	-	Male	Odor of female	Male	5 lux
4 (n = 9)	-	-	+	-	Anosmic male	Anesthetized female	Male	5 lux
5 (n = 11)	-	-	-	+	Anosmic male	Devocalized female	Male	Darkness
6 (n = 10)	+	+	-	-	Male	Odor of female + Playback of USV	Male	5 lux
7 (n = 9)	+	-	+	-	Anosmic male	Anesthetized female + Playback of USV	Male	5 lux
8 (n = 10)	+	-	-	+	Anosmic male	Female	Male	Darkness
9 (n = 10)	-	+	+	-	Male	Anesthetized female	Male	5 lux
10 (n = 11)	-	+	-	+	Male	Devocalized female	Male	Darkness
11 (n = 10)	-	-	+	+	Anosmic male	Devocalized female	Male	5 lux
12 (n = 11)	+	+	+	-	Male	Anesthetized female + Playback of USV	Male	5 lux
13 (n = 11)	+	+	-	+	Male	Female	Male	Darkness
14 (n = 9)	+	-	+	+	Anosmic male	Female	Male	5 lux
15 (n = 10)	-	+	+	+	Male	Devocalized female	Male	5 lux
16 (n = 10)	+	+	+	+	Male	Female	Male	5 lux

An overview of the experimental design of the study. The included (+) and excluded (-) modalities are reported per stimulus group. In addition, it shows whether intact versus anosmic experimental rats were used and what kind of stimulus rat and control rat was placed in the incentive cages. The sexual incentive motivation test was performed under light conditions of 5 lux or complete darkness.

### Behavioral measures and statistical analysis

Sexual incentive motivation was quantified in two ways. First, a *preference score* (time spent in the stimulus incentive zone/ (time spent in the stimulus incentive zone + time spent in the male stimulus incentive zone)) was calculated. Second, the *time spent in the stimulus incentive zone* and *the time spent in the male incentive zone* were used. For comparison between incentives, the preference score as well as the time spent in the vicinity of the incentives should differ in order to consider one incentive as different from another. A double criterion is needed in order to avoid false positive effects. A high preference score could be caused by a very short time spent in the social stimulus zone just as well as by a very long time spent in the stimulus zone. However, a short time spent in the vicinity of the no incentive zone does not necessarily indicate a superior incentive stimulus. Consequently, the chance of false positive differences between incentives is reduced by using a combination of both criteria.

In addition, the number of visits and the latency to enter the incentive zones were evaluated. As indicators of ambulatory activity we employed the total distance moved during the test, the mean velocity of movement while moving, and the time spent moving. These results are described in the supplemental results (S1 File).

The preference score and indices of ambulatory activity were evaluated with an one-factor ANOVA. In case of significance, *a posteriori* comparisons were made with Tukey’s HSD test. The preference score needs to be above .5 (no preference, i.e. the time spent in the incentive zone was equal to the time spent in the no incentive zone) in order to consider that the incentive was efficient. A *t*-test was used for the statistical evaluation. The time spent with the incentives as well as the frequency and the latency to enter the incentive zones was evaluated with two-factor repeated measures (Incentive as within-subject and Type of stimulus as between-subject factor). In case of significance, we performed tests for simple main effects. All probabilities mentioned are two-tailed.

The data was analyzed separately in which the stimulus contained only one modality, two modalities or three modalities. This was always compared to the two control situations in which the incentive stimulus cage was empty or contained an ‘intact’ hormonally primed female.

## Results

### One single-sensory modality does not have more incentive value than social contact

**Fig 1:**
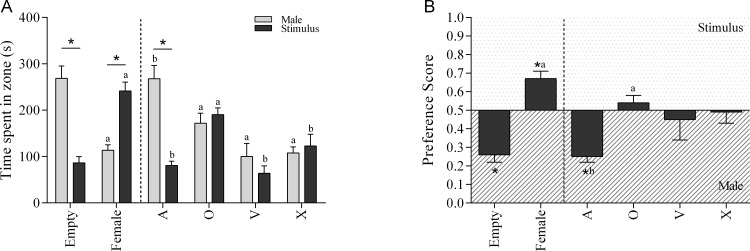
The incentive value assay of a single-sensory stimulus. (A) The time spent in incentive zone and (B) the preference score in the 10-minute sexual incentive motivation test, in which male rats where presented with a single-sensory stimulus of one modality and a control male rat (social stimulus). As control situation an empty incentive cage or a receptive female were presented next to the male rat. A = audition, O = olfaction, V = vision, X = ‘others’. *p<0.05 compared to 0.5 (B) or male rat (A), ^a^p<0.05 compared to ‘empty’ (A, B), ^b^p<0.05 compared to ‘female’ (A, B).

Analysis of time spent in the incentive zone with single-sensory stimuli revealed a significant effect of Type of stimulus (F(5,56) = 14.099, p<0.001) and Incentive (F(1,56) = 8.771, p = 0.004). There was also a significant interaction effect between Type of stimulus and Incentive (F(5,56) = 13.639, p>0.001). Post hoc analysis revealed that the Type of Stimulus effect was caused by a reduction in the time spent in both incentive zones when the experimental males were presented with the single-sensory stimuli vision and ‘others’. In case of the Incentive effect, male rats spent significantly more time with the social stimulus then with an empty box (F(1,56) = 31.50, p<0.001), but they preferred the hormonally primed female compared to the social stimulus (F(1,56) = 14.10, p<0.001) (Fig 1A). In addition, the experimental males spent about the same time nearby the social stimulus and the olfactory, visual, and ‘others’ stimulus (F(1,56) = 0.33, NS; F(1,56) = 0.21, NS; F(1,56) = 1.01, NS, respectively). In the case of the playback of female USVs, the males spent significantly more time in the vicinity of the social stimulus than nearby the auditory stimulus (F(1,56) = 30.08, p<0.001).

Furthermore, one-factor ANOVA analyses of the time spent in the incentive zone of the different single-sensory stimuli revealed that the experimental males only spent significantly more time in the incentive zones of the olfactory stimulus or the hormonally primed female compared to the time spent nearby the empty box (F(5,61) = 16.351, p<0.001). There was no significant difference in time spent in the zone in front of the olfactory stimulus and the hormonally primed female. When comparing the time spent in the vicinity of the social stimulus when the different single-sensory stimuli were presented, it was found that the experimental males spent significantly less time with the social stimulus when a hormonally primed female, or an olfactory, visual, or ‘others’ stimulus was presented (F(5,61) = 12.275, p<0.001). No differences in the time spent with the social stimulus was found during the presentation of an auditory stimulus or an empty stimulus.

Similar results were found in the *t*-test comparing the preference score of the different stimuli with 0.5 (no preference). The empty box (*t*(10) = 6.443, p<0.001), and the auditory stimulus (*t*(9) = 7.942, p<0.001), were less attractive than the social stimulus, whereas a hormonally primed female (*t*(9) = 4.377), p = 0.002) was more attractive than the social stimulus. The olfactory, visual or ‘others’ stimulus abolished the preference for the social stimulus (Fig 1B). Though, only the olfactory stimulus and the hormonally primed female induced a significantly higher preference score compared to the ‘empty’ stimulus, whereas the auditory stimulus was inferior to the hormonally primed female (F(5,61) = 8.061, p<0.01).

In summary, these results show that the presence of a single-sensory stimulus of the modality olfaction, vision, and ‘others’ is sufficient to attenuate the preference for a social contact. However, the single-sensory stimuli do not have a larger incentive value than the social stimulus. This effects was caused by more time the subject males spent with the male rather than an increase in the number of visits (data shown in Text A and Figure A in S1 File). Anosmic males approach both incentives less, and do not distinguish between male and female movements, they do not even care about it, and therefore approach an anesthesized female as much as the moving male (social contact). The auditory stimulus has no effect at all, suggesting that an empty cage with a loudspeaker playing back female USVs was no more attractive than a ‘silent’ empty cage.

### The presentation of a second modality adds incentive value to another modality

**Fig 2:**
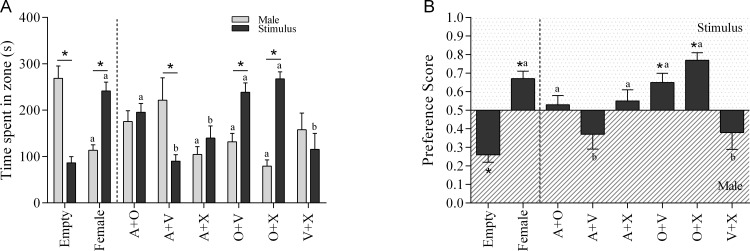
The incentive value assay of a multisensory stimulus of two modalities. (A) The time spent in incentive zone and (B) the preference score in the 10-minute sexual incentive motivation test, in which male rats where presented with a multisensorsensory stimulus of two modalities and a control male rat (social stimulus). As control situation an empty incentive cage or a receptive female were presented next to the male rat. A = audition, O = olfaction, V = vision, and X = ‘others’. *p<0.05 compared to 0.5 (B) or male rat (A), ^a^p<0.05 compared to ‘empty’ (A, B), ^b^p<0.05 compared to ‘female’ (A, B).

To investigate the incentive value of a multisensory stimulus containing two modalities, all possible combinations were investigated with audition, olfaction, vision, and ‘others’. A significant Type of stimulus effect (F(1,72) = 2.906, p = 0.01) and interaction effect of Type of stimulus and Incentive (F(7,72) = 11.549, p<0.001) were found for the time spent in the incentive zones (Fig 2A). Further analysis revealed that besides the control conditions in which the experimental males were presented with an empty box or hormonally primed female on one side and a stimulus male on the other side, the combination of odors and ‘others’ (F(1,72) = 26,25, p<0.001) and odors and vision (F(1,72) = 7,75, p = 0.007) induced more approach behavior towards the stimulus compared to the social stimulus. In addition, it was found that the multisensory stimulus consisting of an auditory stimulus and a visual stimulus had the opposite effect. The experimental males spent more time nearby the social stimulus than this multisensory stimulus (F(1,72) = 10.50, p = 0.002).

Furthermore, one-factor ANOVA analyses of the time spent in the incentive zone of the different multisensory stimuli revealed that the experimental males only spent significantly more time in the incentive zones of the multisensory stimuli compared to spending time nearby the empty box when the stimulus consisted of olfaction in combination with vision, audition, or ‘others’ (F(7,79) = 12.173, p<0.001). In addition, the experimental males spent more time in close vicinity with the hormonally primed female compared to an empty box or a multisensory stimulus containing audition plus ‘others’, audition plus vision, or vision plus ‘others’.

When comparing the time spent in the vicinity of the social stimulus when the different multisensory stimuli were presented, it was found that the experimental males spent significantly less time with the social stimulus when a hormonally primed female, or a multisensory stimulus containing audition plus ‘others’, olfaction plus’others’, or olfaction plus vision was presented (F(7,79) = 6.256, p<0.001).

In terms of the preference score it was found that the multisensory stimuli of olfaction plus ‘others’ (*t*(10) = 6.891, p<0.001) and olfaction plus vision (*t*(9) = 3.247, p = 0.010) induced a significant preference for the stimulus compared to 0.5 (no preference) (Fig 2B). An one-factor ANOVA revealed that all combinations of two modalities in a multisensory stimulus induced a significantly different preference in the experimental rats than the empty stimulus (F(7,79) = 10.140, p<0.01), except for the visual stimulus combined with an auditory stimulus or ‘others’. These multisensory stimuli induced a significantly different preference compared to the hormonally primed female.

In summary, these results show that the combination of two modalities in one multisensory stimulus can have more incentive value than the social contact, as long as one of the modalities is olfactory. The combination of the other modalities without odor are sufficient to attenuate the preference for the social contact without having an adequate enough incentive value to induce preference by itself, like was found with a single-modality stimulus. We can therefore conclude that vision and ‘others’ add value to the olfactory stimulus, when presented together, inducing an incentive value that is similar to an intact receptive female. Again, this effect was caused by an increase in time spent with the stimulus (per visit), instead of an increase in number of visits (data shown in Text B and Figure B in S1 File). Only the combination of olfaction and ‘others’ induced also more visits to the stimulus.

### The presentation of a third modality does not induce more incentive value to other modalities

**Fig 3:**
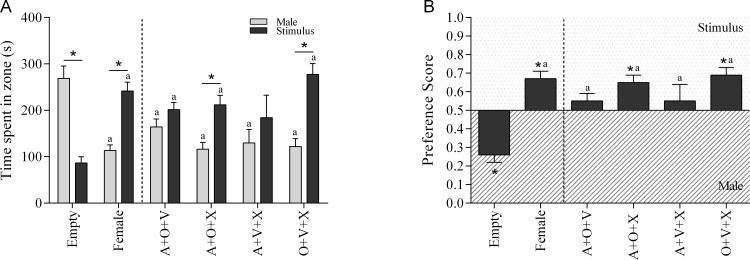
The incentive value assay of a multisensory stimulus of three modalities. (A) The time spent in incentive zone and (B) the preference score in the 10-minute sexual incentive motivation test, in which male rats where presented with a multisensorsensory stimulus of three modalities and a control male rat (social stimulus). As control situation an empty incentive cage or a receptive female were presented next to the male rat. A = audition, O = olfaction, V = vision, and X = ‘others’. *p<0.05 compared to 0.5 (B) or male rat (A), ^a^p<0.05 compared to ‘empty’ (A, B).

The role of a multisensory stimulus including three modalities was also investigated. The results show a significant Incentive effect (F(1,56) = 9.017, p = 0.004) and a significant interaction effect between Type of stimulus and Incentive (F(1,56) = 5,56, p<0.001). As shown in Fig 3A, the multisensory stimulus containing audition, olfaction and ‘others’ (F(1,56) = 6.37, p = 0.014) and the stimulus with olfaction, vision and ‘others’ (F(1,56) = 15.24, p<0.001) induced an increase in time spent in the incentive zone of the multisensory stimulus compared to the time spent nearby the social stimulus.

An one-factor ANOVA revealed that the experimental males spent less time with the social stimulus when a multisensory stimulus or hormonally primed female was presented (F(5,61) = 9.353, p<0.001). In addition, they spent more time with the multisensory stimulus (except for the multisensory stimulus containing an auditory, visual and ‘others’ stimulus) or female than with the empty cage (F(5,61) = 7.378, p<0.001). Again, this effect was not caused by an increase in number of visits to the stimulus (data shown in Text C and Figure C in S1 File).

The same effects were found on the preference score (Fig 3B) in which all multisensory stimuli and the hormonally primed female induced significantly more preference for the stimulus than the social contact (F(5,61) = 10.773, p<0.001). In a *t*-test, the hormonally primed female, the combination of audition, olfaction and ‘others’ (*t*(11) = 3.446, p = 0.006) and the combination of olfaction, vision and ‘others’ (*t*(11) = 4.636, p = 0.001) induced a significant preference for the stimulus compared to 0.5 (no preference).

## Discussion

This study provides evidence that the presentation of one modality can add incentive value to another modality when presented as a multisensory stimulus. A single-sensory stimulus is enough to attenuate the preference for a social contact (except for an auditory stimulus), while the addition of a second modality renders the receptive female more attractive than the social stimulus to a level similar to an intact receptive female. The condition is, however, that the multisensory stimulus contains olfaction in combination with vision or ‘others’ (and not audition in terms of USVs). Adding a third modality, on the other hand, does not change the incentive value of a multisensory stimulus of two modalities.

Our results coincides with an old study by Frank Beach in 1942, in which he investigated the role of multisensory stimuli to elicit mating behavior [25]. Although the actual copulatory behavior follows upon an approach behavior, and belongs therefore to a different phase of sexual behavior, he concluded that “no one of the modalities investigated is essential to copulatory behavior; and at the same time no one is sufficient by itself to arouse excitement leading to mating. The existence of a cooperative function and a resultant summation effect is strongly indicated.” ([25], page 201) This conclusion was drawn from observations that the removal of one modality does not necessarily affect copulatory behavior, while the removal of two or three modalities reduces copulatory behavior. A similar result was also found by Calvin Stone in 1922 [26].

Interestingly, sexually naïve male rats were more severely affected by the removal of multiple modalities than sexually experienced rats [25, 26]. In addition, anosmic rats show impaired copulatory behavior upon their first sexual experience, but when the rats receive sexual experience before the anosmia, the lack of olfactory stimuli does not influence the copulation [27]. This suggests that the role of modalities in sexual behavior changes with sexual experience. The males used in our study were sexually experienced before the SIM test, and should therefore be familiar with the meaning of the different modalities in the sexual context.

In addition, our results confirm our previous findings in which an *olfactory stimulus* has incentive value, while the playback of ultrasonic vocalizations (USVs) did not induce approach behavior in rats [10, 16]. The observation that the odor of a hormonally primed female in the current study is only sufficient to attenuate the preference for a social stimulus, while inducing preference in the previous study, can be explained by the fact that in the prior experiment an empty incentive cage was presented as control condition instead of a social stimulus [10, 16]. This suggests that the social stimulus in the current experiment competes with the (multi)sensory stimuli. The presentation of a single-sensory stimulus of olfaction, vision or ‘others’ changes the preference for the social stimulus into an equal interest. The benefit of this set-up is that it was possible to find the additional value of a second (or third) modality in a multisensory stimulus, because ceiling effects did no longer limit the readout of the results.

The current data clearly show the importance of olfaction in an initial approach behavior towards a potential mate. The single-sensory stimuli with vision and ‘others’ are also capable of attenuating the preference for a social contact, but a clear preference to the level of an intact receptive female is only induced by a multisensory stimulus that contains olfaction. The combination of vision and ‘others’ did not have more incentive value than the social contact. Only the olfactory stimulus induced a significant increase in approach behavior towards the single-sensory stimulus compared to the control situation of the empty cage. When the stimulus contained only vision or ‘others’, the anosmic experimental male approached both the single-sensory stimulus and the social stimulus less. They could no longer distinguish between males and females and thereby lost their interest; an anesthesized female was even approached as much as the moving male. The important role of olfaction in approach behavior has been shown many times [8–11, 28–31], just as its significance in sexual behavior [27, 31]. It is therefore not surprising that olfaction turns out to play an important role in the incentive value of a (multisensory) stimulus, which indicates that olfaction is an important factor in the attractiveness of a receptive female.

The observation that a *visual stimulus* by itself is able to attenuate the preference for a social stimulus, on the other hand, is surprising. Previously, it has been shown that males approach females also in complete darkness [6, 7] and copulate in a normal manner when surgically blinded [25, 32]. This suggests that vision is not essential in sexual behavior and mate selection. However, our results show that vision is actually an important modality in approach behavior towards a potential mate. The presence of an anesthesized hormonally primed female by itself, without her odors,vocalizations or ‘others’, reduces the interest for the social stimulus and in combination with the smell of a receptive female, the multisensory stimulus is prefered over the social stimulus to a level similar of an intact, receptive female. It should be mentioned, though, that these results are actually similar to the previous observation in which it was concluded that vision does not play an important role. In those studies the rats were still able to smell the females, and thus they have not studied vision alone, but always in combination with olfaction. The current study systematically investigated the role of vision, both in the absence and in the presence of other modalities. The data showed that the combination of vision and audition or ‘others’ did not have an effect on the incentive value of the (multisensory) stimulus. Therefore, we conclude that although visual stimuli from the hormonally primed female are not essential for sexual approach behavior, they can attract the experimental male by itself and add incentive value to the modality of olfaction.

The other modality that is able to attenuate the preference for the social contact by itself and adds incentive value to olfaction is *‘others’*. It is a modality that has not really been investigated before, because it is difficult to describe ‘others’. As mentioned before, the ‘modality’ ‘others’ in this study should be interpreted as the other factors that could be involved in approach behavior, besides the emitted ultrasonic vocalizations, the smell of a hormonally primed female and the sight of a female. It is so far unclear what they exactly represent, but they could be for instance the sound of a moving or darting female, the smell of a rat being closer or more at distant, or the feeling of a temperature change or a flow in the air generated by the body of a moving rat. In addition, it could be that a darting female induces vibrations of the floor that can reach the male and persuade him to approach the stimulus. There are possibly other factors that could be added to this category, but which are not distinguished in this (and other) studies. Interestingly, the results show that the combination of these ‘other’ characteristics are important in the incentive value of a female. The single-sensory stimulus of these ‘others’ by itself, with the exclusion of olfaction, vision and audition (USVs), is able to reduce the interest for the social stimulus. The combination of this ‘others’ with the smell of a receptive female even induces preference for the multisensory stimulus. This suggests that we have always missed a factor that is involved in inducing approach behavior. Although our study does not conclude on what this factor exactly is, it does show the importance of future research on the incentive value of the nonobvious characteristics of a moving animal.

The only modality that did not induce additional incentive value as multisensory stimulus is *audition*. As mentioned before, our results coincide with previous studies that show that USVs do not have an incentive value for male and female rats [10, 16]. Therefore, it is not surprising that the playback of USVs by itself does not induce approach behavior or attenuate the preference for the social contact. However, the current data also shows that USVs do not play an additional role to e.g. olfaction. The supplementation of audition as third modality to vision and olfaction does even reduce the incentive value of vision and olfaction together. This would suggest that the playback of vocalizations in addition to olfaction and vision would have a negative effect on the incentive value of the multisensory stimulus.However, since there is no logical explanation for this finding it could also be an artifact. Still, the limited role for USVs in approach to a potential mate is also supported by previous studies in which rats are tested in a multiple partner set-up; silent conspecifics were as often and as long visited as vocalizing rats [4, 5]. Also in a seminatural environment, in which USVs could in theory function to induce the attention of a potential mate at a long distance, silent females did receive the similar amount of sexual interactions as vocalizing females [33]. The same results were found in normal copulation tests with devocalized [34–37] or deafened [25] rats. All together, we conclude that USVs are not relevant in approach to a potential mate or sexual behavior.

At last, it should be mentioned that in general it seems that anosmia induces lower ambulatory activity in the experimental rats (see Table A in S1 File). However, this reduction in locomotor activity did not affect the other parameters like the number of visits to each incentive zone or latency to the first visit, suggesting that the lower ambulatory activity did not influence the approach behavior towards the (multi)sensory stimuli. The benefit of the SIM test over other tests used to study motivation is that it does not employ learned operant responses like running in a runway or bar pressing for access to a mate (e.g. [38, 39]). These responses can easily be mistaken for effects of learning or memory of the procedure, but more significantly, the rate or speed of responding is an important factor in operant procedures and could severely affect the motivational read-out. The SIM test, on the other hand, employs permanence in a particular area as an index of motivation, minimizing the requirement of motor capacities. The lower ambulatory activity in anosmic rats, therefore, does not change our conclusions about the incentive value of the different (multi)sensory stimuli.

All together, we can conclude that the loss of a single modality is not disastrous for sexual selection, while the loss of two modalities can affect the initial approach behavior when olfaction is one of them. Frank Beach’ suggestion of the existence of a cooperative function and a resultant summation of modalities is strongly supported by the data. A receptive female rat can attract a conspecific by her smell, vision and ‘others’ alone, but the combination of these factors increases the attractiveness of the female. The potential mates with the highest attractiveness have, from a biological perspective, a considerable reproductive advantage over the others. The initial approach behavior seems to be induced by the combination of at least two modalities from which olfaction is crucial. Future studies investigating the biological mechanisms behind approach behavior should focus on the brain areas involved in the interpretation of olfactory stimuli and the integration of this information with additional sensory stimuli like vision and b the sofar undefined ‘others’.

## Supporting information

S1 File Supplemental result. (DOCX)
