# Supplementary material for: A cooperative function for multisensory stimuli in the induction of approach behavior of a potential mate
Source: PLoS One. 2017 Mar 17;12(3):e0174339. doi: 10.1371/journal.pone.0174339 (PMC5357056; doi:10.1371/journal.pone.0174339)
Supplement: S1 File — (DOCX) [file pone.0174339.s001.docx]

**S1 File. Supplemental Results**

**A cooperative function for multisensory stimuli in the induction of approach behavior of a potential mate**

**Anders Ågmo^1^ and Eelke MS Snoeren^1*^**

^1^ Department of Psychology, UiT The Arctic University of Norway, Tromsø, Norway

* Correspondence: [eelke.snoeren@uit.no](mailto:eelke.snoeren@uit.no)

**Text A. The number of visits and latency to visit of a single-sensory modality**

No significant effect of Type of stimulus or Incentive was found for the number of visits to the incentive zones, just as no interaction effect of Type of stimulus and Incentive (Fig S1a). However, a test for simple main effects indicated that the experimental males visited the male stimulus more often than the empty (F(1,56)=7.86, p=0.007) and the auditory stimulus (F(1,56)=4.33), p=0.042). Only when a visual stimulus was presented, the experimental males visited the social contact more often than when an ‘empty’ stimulus was present (F(5,61)=2.659, p=0.032). Furthermore, there was no Type of stimulus effect on the latency to the first visit to the incentive zones. However, there was a significant Incentive effect (F(1,56)=4.444, p=0.04) and interaction effect of Incentive and Type of stimulus (F(5,56)=3.266, p=0.012) on the latency to the first visit to the incentive zones (F(1,56)=4.444, p=0.04). Post hoc analysis revealed that this effect was only caused by an unexplainable, long latency to visit the social stimulus compared to the visual stimulus (Fig S1b). One-factor ANOVA also revealed a significant effect of the time spent nearby the social stimulus when presented with the visual stimulus compared to the situation in which the ‘empty’ stimulus or hormonally primed female were present (F(5,61)=3.325, p=0.011).

**Fig A. The number of visits and latency to visit of a single-sensory stimulus**


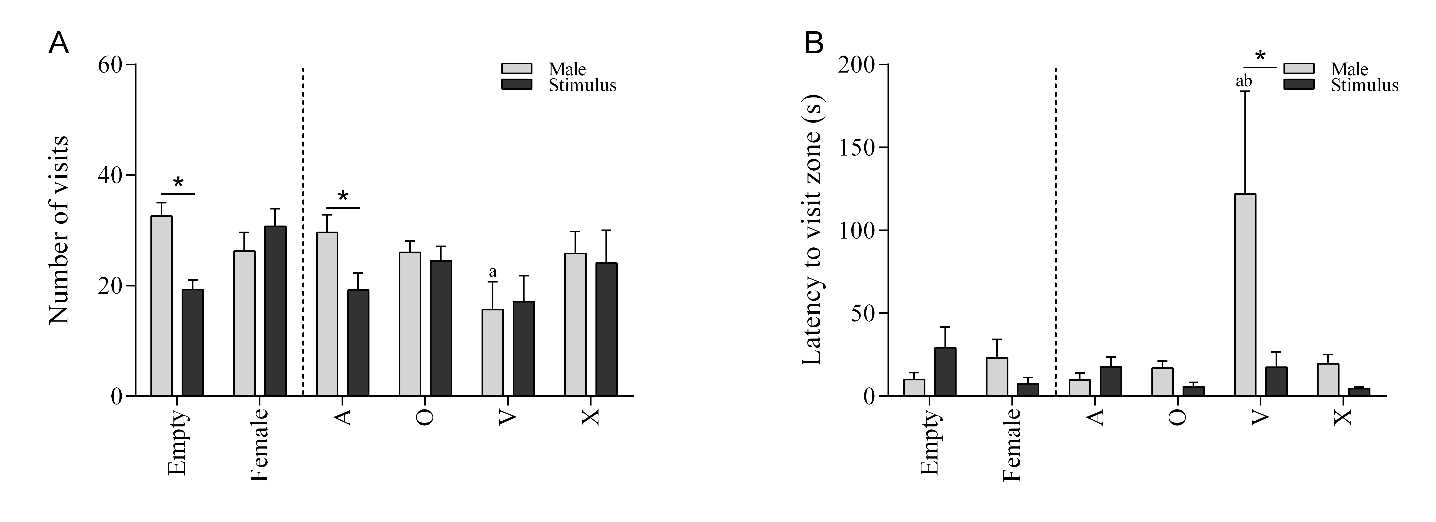
 (A) the number of visits to the incentive zones, and (B) the latency to the first visit of each incentive zone in the 10-minute sexual incentive motivation test, in which male rats where presented with a single-sensory stimulus of one modality and a control male rat (social stimulus). As control situation an empty incentive cage or a receptive female were presented next to the male rat. A = audition, O = olfaction, V = vision, X = ‘others’. *p<0.05 compared to male rat (A, B), ^a^p<0.05 compared to ‘empty’ (A, B), ^b^p<0.05 compared to ‘female’ (A, B).

**Text B. The number of visits and latency to visit of a multisensory stimulus of two modalities.**

A significant interaction effect between Type of stimulus and Incentive was found (F(7,72)=3.964, p=0.001) for the number of visits to the incentive zones. No difference was found for the latency to first visit (Fig S2). The tests for simple main effects showed that only the multisensory stimulus of olfaction and ‘others’ induced more visits to the incentive zone than the social stimulus (F(1,72)=7.13, p=0.009). In response to the multisensory stimulus with vision and ‘others’, the rats visited the social stimulus significantly faster (F(1,72)=5.79, p=0.019) and more often (F(1,72)=14.69, p>0.001) than the multisensory stimulus of vision and ‘others’. However, only with the multisensory stimulus of olfaction and ‘others’, the experimental males visited the incentive zone of the stimulus significantly more often than the incentive zone of the empty cage (F(7,79)=3.568, p=0.002).

**Fig B. The number of visits and latency to visit of a multisensory stimulus of two modalities**


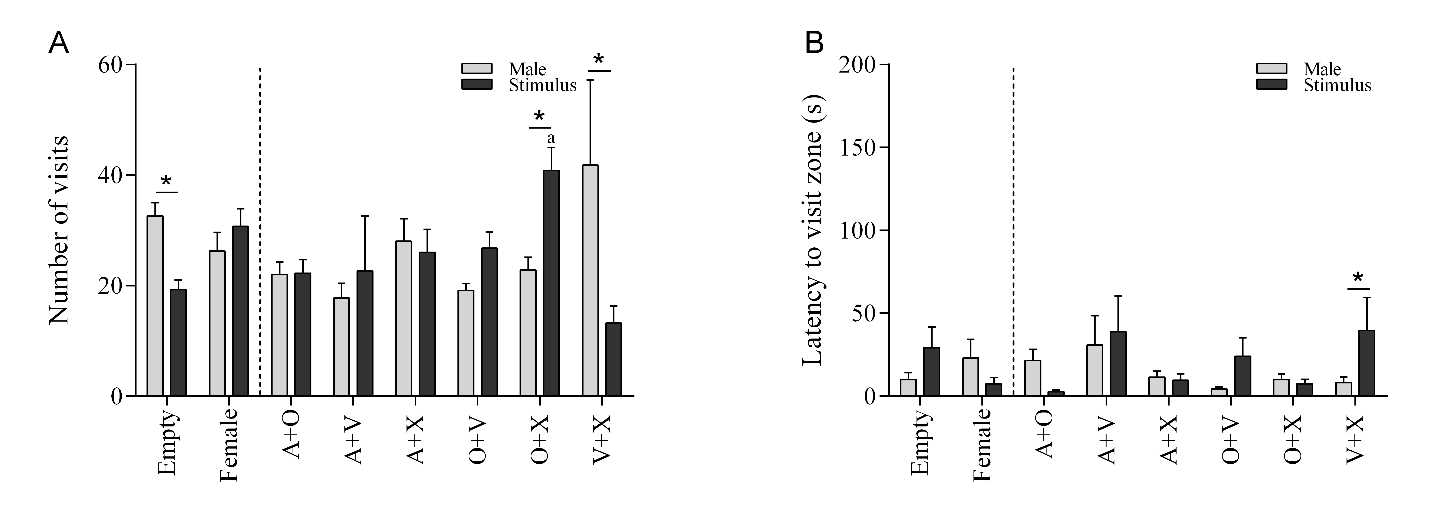
(A) the number of visits to the incentive zones, and (B) the latency to the first visit of each incentive zone in the 10-minute sexual incentive motivation test, in which male rats where presented with a multisensorsensory stimulus of two modalities and a control male rat (social stimulus). As control situation an empty incentive cage or a receptive female were presented next to the male rat. A = audition, O = olfaction, V = vision, X = ‘others’. *p<0.05 compared to male rat (A, B), ^a^p<0.05 compared to ‘empty’ (A, B), ^b^p<0.05 compared to ‘female’ (A, B).

**Text C. The number of visits and latency to visit of a multisensory stimulus of three modalities.**

In the number of visits to the incentive zones (Fig S3a), only a significant interaction effect of Type of stimulus and Incentive was found (F(5,56)=3.519, p=0.008). This effect was probably caused by a lower number of visits to the multisensory stimulus containing audition, vision and ‘others’ in comparison to the social stimulus (F(1,56)=4,25, p=0.044). Only when a hormonally primed female was presented, the experimental male visited the stimulus more often than the empty cage (F(5,61)=3.452, p=0.009). No differences were found on the latency to the first visit (Fig S3b).

**Fig C. The number of visits and latency to visit of a multisensory stimulus of three modalities**


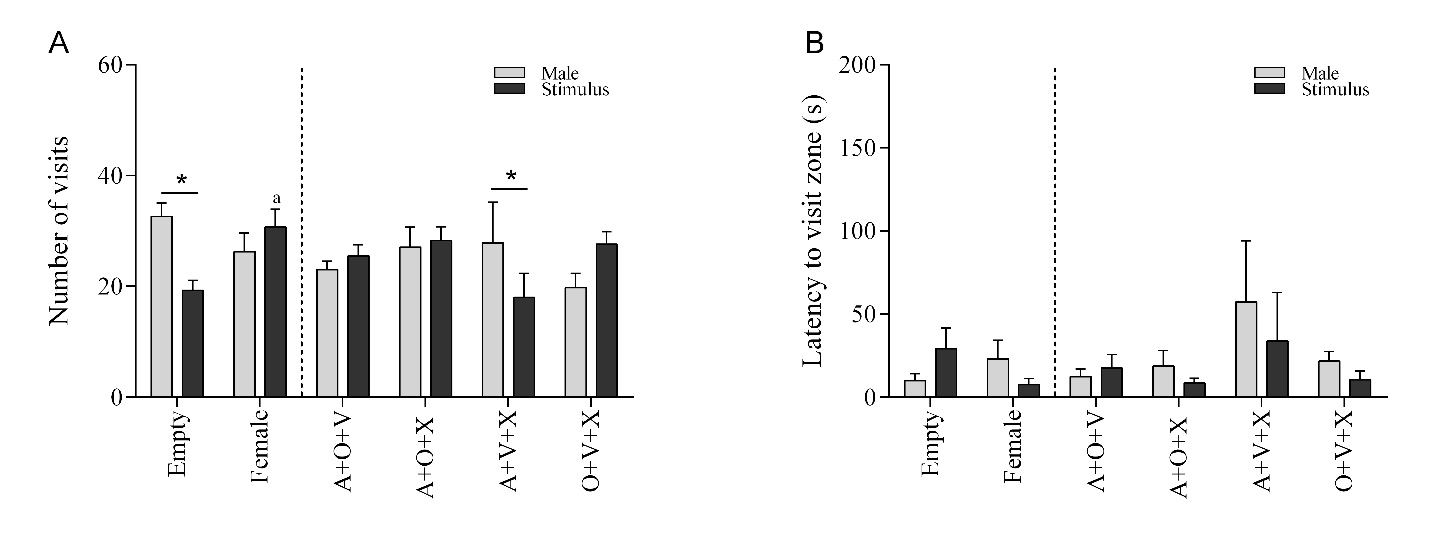


(A) the number of visits to the incentive zones, and (B) the latency to the first visit of each incentive zone in the 10-minute sexual incentive motivation test, in which male rats where presented with a multisensorsensory stimulus of three modalities and a control male rat (social stimulus). As control situation an empty incentive cage or a receptive female were presented next to the male rat. A = audition, O = olfaction, V = vision, X = ‘others’. *p<0.05 compared to male rat (A, B), ^a^p<0.05 compared to ‘empty’ (A, B), ^b^p<0.05 compared to ‘female’ (A, B).

**Table A. Ambulatory activity in response to different (multi)sensory stimuli**

| Stimulus group | (multi)sensory stimulus | Movement (s) | Velocity (cm/s) | Distance moved (cm) |
| --- | --- | --- | --- | --- |
| 1 (n=11) | Empty | 438.22±9.96 | 8.00±0.48 | 4789.76±284.93 |
| 2 (n=10) | A | 476.34±7.97 | 7.92±0.58 | 4740.95±343.01 |
| 3 (n=11) | O | 459.71±6.02 | 8.25±0.35 | 4937.42±212.26 |
| 4 (n=9) | V | 342.89±25.70*# | 4.40±0.54*# | 2635.94±320.97*# |
| 5 (n=11) | X | 457.67±24.52 | 8.04±0.76 | 4811.11±451.82 |
| 6 (n=10) | A+O | 455.11±9.52 | 7.69±0.54 | 4601.69±320.41 |
| 7 (n=9) | A+V | 400.19±20.37# | 5.60±0.44*# | 3356.77±261.21*# |
| 8 (n=10) | A+X | 513.38±13.57* | 9.16±0.56 | 5477.86±330.49 |
| 9 (n=10) | O+V | 441.31±9.03 | 7.30±0.35 | 4371.08±208.55 |
| 10 (n=11) | O+X | 520.10±10.13*# | 11.01±0.47* | 6589.84±279.96* |
| 11 (n=10) | V+X | 408.55±18.34 | 5.72±0.51*# | 3426.28±303.04* |
| 12 (n=11) | A+O+V | 460.74±10.69 | 8.16±0.32 | 4881.10±192.20 |
| 13 (n=11) | A+O+X | 512.88±13.65 | 9.52±0.38 | 5708.48±225.46 |
| 14 (n=9) | A+V+X | 398.32±21.30# | 5.79±0.49*# | 3471.36±295.73*# |
| 15 (n=10) | O+V+X | 456.33±8.01 | 7.83±0.36 | 4681.92±217.74 |
| 16 (n=10) | Female | 462.68±7.47 | 9.03±0.61 | 5406.17±362.90 |

Ambulatory activity measured by movement (s), velocity (cm/s) and distance moved (cm) in the 10-minute sexual incentive motivation test. Male rats where presented with a single or multisensory stimulus and a control male rat (social stimulus). As control situation an empty incentive cage or a receptive female were presented next to the male rat. A = audition, O = olfaction, V = vision, and X = ‘others’. *p<0.05 compared to the empty “stimulus” and #p<0.005 compared to the female stimulus.
